# Global collaborative research in metabolic and bariatric surgery (GCRMBS): current status and directions for the future

**DOI:** 10.1186/s12893-024-02636-4

**Published:** 2024-11-20

**Authors:** Sjaak Pouwels, Omar Thaher, Miljana Vladimirov, Daniel Moritz Felsenreich, Beniamino Pascotto, Safwan Taha, Dirk Bausch, Rodolfo J. Oviedo

**Affiliations:** 1 Department of Surgery, Marien Hospital Herne, University Hospital of Ruhr University Bochum, Hölkeskampring 40, 44625 Herne, NRW Germany; 2 Department of Intensive Care Medicine, Elisabeth-Tweesteden Hospital, Tilburg, The Netherlands; 3 Department of Surgery, University of Bielefeld – Campus Detmold, Detmold, NRW Germany; 4 Department of Surgery, Vienna Medical University, Vienna, Austria; 5 Department of General and Minimally Invasive Surgery, Centre Hospitalier de Luxembourg, Luxembourg, Luxembourg; 6 Department of Metabolic and Bariatric Surgery, Mediclinic Airport Road Hospital, Abu Dhabi, United Arab Emirates; 7 Nacogdoches Medical Center, Nacogdoches, TX USA; 8 University of Houston Tilman J. Fertitta Family College of Medicine, Houston, TX USA; 9 Sam Houston State University College of Osteopathic Medicine, Conroe, TX USA

**Keywords:** Research, Bariatric Surgery, Metabolic Surgery, Collaboration, Obesity

## Abstract

Obesity has been recognized as a chronic disorder by the World Health Organisation (WHO) and was first reported in the Paleolithic age. In the recent years there has not been an international collaborative that facilitates professional cooperation on a worldwide level to increase the output of high-level evidence in the fields of obesity treatment and metabolic and bariatric surgery (MBS). In other surgical and medical fields, international collaborative research networks have shown to increase the quality and amount of treatment-changing evidence. In general, Global Collaborative Research in MBS (GCRMBS) should have the following goals: (1) clinical specialty–based research in obesity and MBS, (2) designing research protocols and studies to generate long-term data in obesity and MBS, (3) understanding the uncommon/rare complications and events associated with obesity and MBS, (4) increasing the number of participants in research and (5) investigating ethical and racial disparities in bariatric research. This review gives an overview of the current status and the future of international collaborative research in MBS.

## Introduction

The World Health Organisation (WHO) recognized obesity has a chronic disorder [1]. Nowadays obesity has become one of the major health challenges and has gained increased interest since the COVID-19 pandemic due to the fact that obesity is a major risk factor for a complicated course of COVID-19 infection [2]. Data from the WHO and several population-based studies indicate that more individuals throughout the world are becoming overweight or suffering from obesity [3, 4]. Worldwide, there is a rise of obesity in the last fifty years, which corresponds with worrisome numbers. Global estimates indicate that over 1.9 billion adults are overweight and 650 million adults have obesity among them [5, 6]. This is a rise of 300% [5, 6].

Moreover, the world human population has also increased, indicating that the prevalence of obesity is around 37% compared to 27% in 1980 [5, 6]. Obesity increases the morbidity and mortality risk for several diseases such as type 2 diabetes mellitus (T2DM), obstructive sleep apnoea syndrome (OSAS), fatty liver disease, cardiovascular diseases (CVD) and metabolic syndrome [7–10]. Therefore, the American Medical Association (AMA) and the WHO stated that obesity is considered a chronic disease and not solely a risk factor for other related diseases [3, 11]. On the other hand, the rise in the incidence of obesity that seen a comparable rise in medical research in the field of obesity treatment. This was pointed out by a bibliometric analysis done by Zhao et al. [12] which showed that between 1999 and 2017 the cumulative number of publications in the field of obesity followed an exponential growth pattern (R^2^ = 0.9974). The United States of America was the most prolific country in international papers, and the two most prolific journals were Obesity Surgery and the International Journal of Obesity (together responsible for 3.95% of all publications [12]. Interestingly, among the top prolific countries the majority are European. In other fields, collaborative research networks have contributed to increase the level and amount of treatment-changing evidence [13–15]. Our study gives an overview of frequently encountered problems in medical research and which aspects should be prioritized based on large-scale collaborative research in MBS.

## General problems in current medical research

The evidence generated from clinical research is considered the backbone of modern medicine. Well-designed high-quality studies are meant to inform healthcare policy, influence medical decision-making and stimulate quality improvement [16]. In the evidence-based era we live in, the importance of high-quality studies cannot be understated or underestimated. This is because the majority of our national and international protocols are based on these studies. A good example is that the evidence from clinical trials can be used to justify reimbursement for healthcare and services [17]. Depending on the country, regulatory agencies determine which medical/surgical treatment deserves reimbursement and also how much of the treatment will be reimbursed [16–18].

Despite a growing demand for high-quality studies, worldwide clinical research output is facing new challenges in addition to the ones arising from the COVID-19 pandemic [2, 18, 19]. One of the major aspects of clinical trials is funding. To design a decent (randomised) clinical trial, the costs can be a major challenge and their acquisition has become a very competitive task [19]. This is compounded by a shrinking clinical investigator workforce, especially in the smaller and non-academic hospitals [15, 18, 20]. In the United States there is a combination of low amount of clinical investigators and a particularly high turnover of research personnel [18–20].

It also has to be taken into account that all trials conducted place a demand on the general public to participate. Other problems that arise are that clinical trials do not meet their enrolment deadlines [21] and often do not deliver information that is valuable to clinicians and patients [22]. Clinical trials that rely on surrogate markers and laboratory values might be able to be translated to clinical practice [22]. Research in surgical specialities encounter obstacles of their own that limit research output [23]. The main requirement for a randomised clinical trial is clinical equipoise, a general uncertainty to recommend one treatment over another. However, patients and clinicians might a have preference for one treatment modality (e.g. surgery) which can increase difficulties to include patients in trials. In many surgical trials, blinding is not possible or in some cases it may even be unethical. Furthermore, differences in surgeon skill can introduce more complexity to conducting surgical trials [23, 24]. This is especially the case when examining outcomes (complications and mortality), which can be skewed since favourable outcomes are achieved on the basis of skill relative to other surgeons [18, 23–25]. Finally, when studying newer surgical techniques a learning curve phenomenon can be encountered [23].

There is a change necessary in the organisation of clinical research in MBS to overcome systemic and specialty-specific factors that impede the development of high-quality evidence. Single centre studies conducted by one primary investigator have been the mainstay of clinical research in MBS for a long-time, yet this may contribute to inefficiency and poor-quality research output. Recent initiatives like the GENEVA and ONWARD studies [26, 27] can be a good example of the future of clinical research in MBS.

**Fig. 1:**
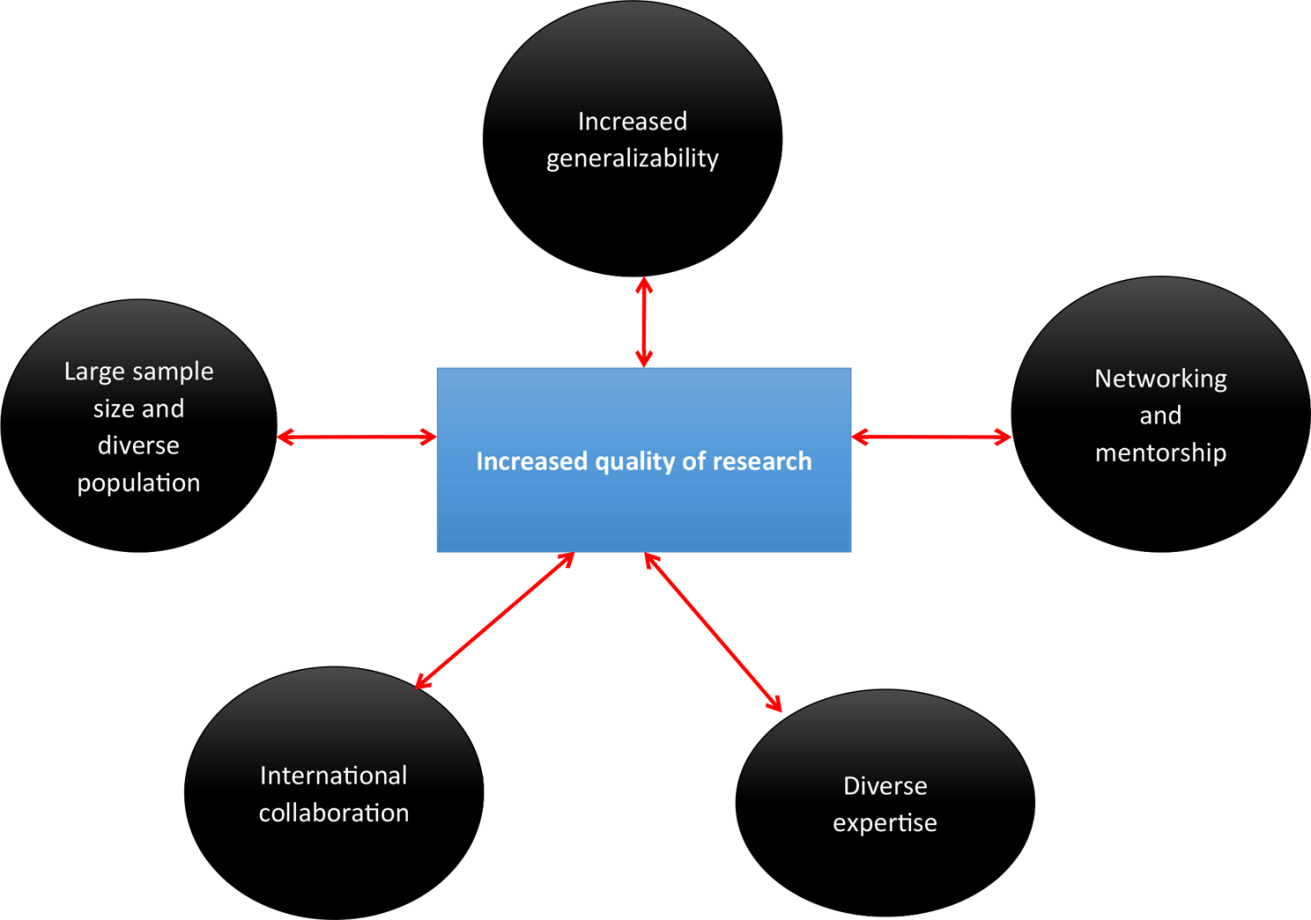
Characteristics of multicentre studies

The procurement of funding for multicentre studies is the one of the first steps towards a more productive research enterprise, especially if studies are performed within an international collaborative group (Fig. 1). Firstly, these international multicentre studies are enriched by the expertise of a diverse group of clinical investigators, expediting the development of research protocols and trials. This will eventually lead to a faster dissemination of evidence [25]. Secondly, these collaborations permit larger sample sizes drawn from heterogeneous patient groups, which will increase the generalizability of the study results [15, 25, 28]. Thirdly, these multicentre collaborations will decrease the study work burden and will decrease the inefficient one-off paradigm, a pattern where resources and personnel align for the period of the trial and afterwards are dissolved [15, 18, 20, 28].

## Current problems in research in metabolic and bariatric surgery

The obesity pandemic was a growing problem worldwide and got even more attention because of the COVID-19 pandemic, because several studies pointed out that obesity is an independent risk factor for COVID-19 [2, 5]. MBS is still the most effective long-term treatment for obesity. The National Institutes of Health (NIH) consensus statement published 22 years ago largely governed the use of MBS worldwide [29]. Per such consensus document’s recommendations, patients with a body mass index (BMI) greater than 40 kg/m^2^, or greater than 35 kg/m^2^ with obesity-related diseases such as type 2 diabetes, are eligible for MBS. Although these recommendations were carefully written and based on evidence (available at that time) they are outdated and have important limitations in the current era [30, 31]. This resulted in the recently newly published guidelines of the International Federation of Surgery of Obesity and Metabolic Disorders (IFSO) [32].

Even after these guidelines were published, not every country has implemented them (yet). In 2021 this NIH statement was still used as a guideline, but the surgical treatment for obesity is shifting to a more metabolic (rather than a pure bariatric) approach. One of the main points is a more physiological approach to the surgery itself, naming it metabolic surgery or ‘bariatric and metabolic surgery. ’ Scientific literature showed that it is more apparent to tailor the surgical procedure to the metabolic profile of patients. MBS has shown pivotal effects on on metabolic diseases, such as type 2 diabetes mellitus (T2DM) [31, 33–36], but also pulmonary and cardiac diseases, like asthma [37, 38] and congestive heart failure [39, 40]. The well-known landmark study in MBS, the Swedish Obese Subjects study has shown that MBS is capable of major reductions in cardiovascular risk factors and events, and even mortality [41]. Therefore the new IFSO guidelines broadened the indications for MBS [32]. 

With increasing understanding of the physiology of T2DM remission, more insights have been gained regarding other physiologic changes. One of these is the prevalence of nutrient deficiencies and how to optimise a patient prior to specific MBS procedures [42–44], but also the body of literature regarding cardiac [45] and pulmonary physiology [37, 38, 46] after MBS is growing. Moreover, the physiologic mechanisms of MBS on the amelioration of type 2 diabetes, cardiovascular and pulmonary diseases are thought to be weight-dependent and also weight independent [41].

Interestingly, with the above-mentioned changes and increasing interest in the physiology of heart and lung disease after MBS, significant evidence has been generated on these subjects. For example, a PubMed search on “heart failure and bariatric surgery” shows 275 results, and “asthma and bariatric surgery” shows 167 results compared to 7,413 results on “diabetes mellitus and bariatric surgery.”

In particular, in patients with cardiac pathology and obesity, it seems that MBS is beneficial for myocardial structure, systolic and diastolic function [47]. Several case series have demonstrated the positive effect of MBS on left ventricular ejection fraction (LVEF) and the New York Heart Association (NYHA) functional class (of heart failure) [48–52]. Ristow et al. [53] reported on two patients who no longer required heart transplantation after successful weight reduction and improvement of LVEF. In summary, to change current treatment guidelines more high-quality evidence must be generated for patients with cardiac pathology, but also for other patients with organ disease (e.g. failure) from severe obesity. To do this there is a need for high quality, preferably multicentre, studies.

## Recommendations for future multicentre collaborative research

Four main goals for research in the next few years: (1) clinical specialty–based research in obesity and MBS, (2) designing research protocols and studies with the purpose of generating long-term data in obesity and MBS, (3) understanding the uncommon/rare complications and events associated with obesity and MBS, (4) increasing the number of participants in research and (5) investigating ethical and racial disparities in bariatric research.

### Speciality driven research versus operation-based research

**Fig. 2:**
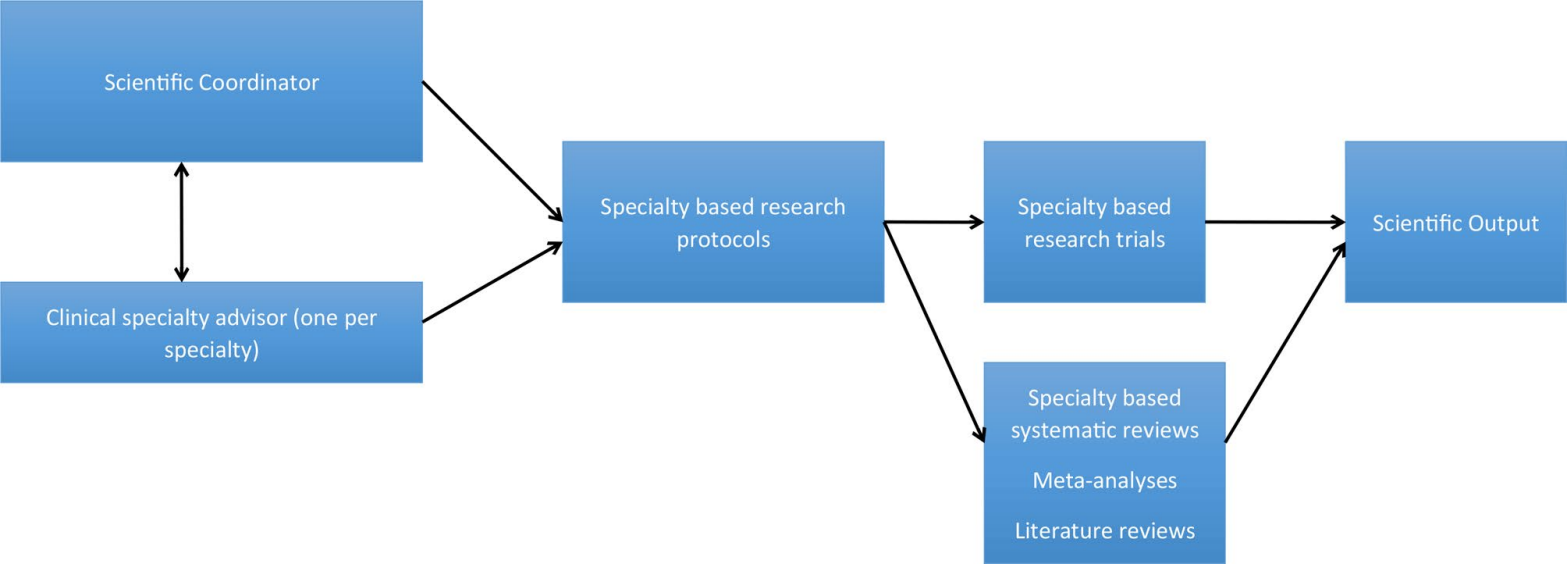
Proposed structure of GCRMBS specialty-based research

Figure 2 gives an overview of the structure of specialty-based research. This means that in collaborative research there is need for structuring research in obesity and MBS. For example, in every specialty-based research there needs to a clinical specialty advisor in the form of a consultant of the specialty in particular. He or she will coordinate the research efforts within the given specialty group, will design trials and systematic reviews with the specialty group, and will communicate these strategies and research plan to the scientific coordinator. The scientific coordinator will in turn oversee all the research tasks within the specialty subgroups. Eventually this will lead to research groups within all the specialties mentioned in Table 1.

**Table 1:** List of medical specialties for specialty-driven research

Medical specialties	Surgical specialties	Allied health
Anaesthesiology	Cardiothoracic Surgery	Psychology
Cardiology	Otorhinolaryngology	Nursing
Dermatology	Urology	Physical Therapy
Internal Medicine	Plastic Surgery	Nutrition
Genetics	Ophthalmology	
Paediatrics	Orthopaedic Surgery	
Geriatrics	Neurosurgery	
Pulmonology	General Surgery and subspecialties	
Gastroenterology	Obstetrics and Gynaecology	
Microbiology		
Neurology		
Radiology and Nuclear Medicine		
Oncology		
Pathology		
Psychiatry		
Radiotherapy		
Rheumatology		
Rehabilitation Medicine		
Emergency Medicine		
Sports Medicine		
Primary Care		
Occupational Health		
Intensive Care Medicine		

### Long-term data

The second future goal should be to generate evidence showing long-term data in obesity treatment and MBS. Nowadays, more long-term data are arising from separate types of MBS procedures. Recently, Mingrone et al. [54] published the 10-year results of a RCT comparing medical therapy with MBS (RYGB or BPD) on T2DM remission. They concluded that MBS has a significant long-term effect on T2DM remission.

However, for other organ diseases like cardiac and lung pathology this evidence is lacking. Therefore, the goal of our proposal for Global Collaborative Research in Metabolic and Bariatric Surgery (GCRMBS) is to make high-quality multicentre studies to investigate the long-term effects of MBS on organ diseases. One of the first attempts in this particular goal is to evaluate the effects of MBS on cirrhosis, which has been recently published as a systematic review [55].

### Understanding uncommon/rare complications after metabolic and bariatric surgery

We should be able to understand the more uncommon/rare events and complications in obesity treatment and after MBS. Recently our group published a systematic review on thoracic fistulae after sleeve gastrectomy and we proposed a treatment algorithm for this difficult surgical situation [56]. Zhang et al. [57] reported on hair loss after MBS, which is an uncommonly seen problem. They concluded that according to current evidence it is more often seen in younger women and associated with low serum levels of zinc, folic acid and ferritin. Future research should focus on increasing our understanding of rare complications after MBS to optimize patient care, quality of life and well-being [56, 58–61]. 

### Investigating ethical and racial disparities in obesity and bariatric research

In the last few years, more attention in treatment and research has become on possible ethical and racial disparities in obesity and bariatric research. One of them is the different effects in for example the Asian population and possible different effects in Low-BMI groups. Also access to obesity treatment and bariatric and metabolic surgery should be part of an international collaborative [56, 58–61]. 

## Conclusion

Multicentre collaborative trials offer solutions to various problems such as high-quality methodology, increased sample size, enhancing generalizability and diversifying the representation of patients and practice settings. Potential downsizes like reporting bias, differences in surgical techniques and other potential confounders can be reduced in large-scale multicentre collaborative studies with clear research questions and/or objectives. The earlier mentioned goals will increase specialty-based obesity and MBS research, providing long-term data and analysing uncommon and rare complications in this field. The proposed GCRMBS model will provide the research infrastructure for successful multicentre collaborative studies that will facilitate much needed guideline changes.

## Data Availability

No datasets were generated or analysed during the current study.
